# New insight into the pigmented rice of northeast India revealed high antioxidant and mineral compositions for better human health

**DOI:** 10.1016/j.heliyon.2022.e10464

**Published:** 2022-08-28

**Authors:** Sagolshem Priyokumar Singh, Vanlalsanga, S.K. Mehta, Y. Tunginba Singh

**Affiliations:** Department of Botany, Mizoram University, Aizawl, 796004, Mizoram, India

**Keywords:** Northeast India, Pigmented rice, Antioxidants, Minerals, FTIR

## Abstract

Northeast (NE) India possesses a rich diversity of rice cultivars including pigmented and non pigmented varieties. The pigmented rice is reported to possess a considerable amount of antioxidant compounds, free radical scavengers etc. In this study, eleven (black, red and white) rice cultivars of NE India were analyzed for antioxidant potentials, mineral and protein contents. Total phenolic content ranged from 94.8 (Idaw) to 900.90 mg GAE/100 g (Lumre). Total flavonoid content varied from 3.46 (Idaw) to 286.76 mg QE/100 g (Menil mibabaret). Total anthocyanin content varied from 0.23 (Farel) to 93.52 mg/100 g (Chakhao poireiton). The pigmented rice is also good sources of Catalase (CAT), Ascorbate peroxidase (APX) and Superoxide Dismutase (SOD) that can significantly reduce stress oxidative reactions. Chakhao poireiton possessed the highest Ni and Mn content, Tsulu tsuk had the highest Zn content, while Fazu and Tasung contained the highest Fe and Ca. The highest total protein was found in Chakhao poireiton (11.06%). And all the cultivars were found to be aromatic. Fourier Transformed Infra-Red spectroscopy (FTIR) identified various signature peaks and could discriminate the cultivars into pigmented and non pigmented. Principle Component Analysis (PCA) revealed the grouping of the cultivars based on the functional groups present. The present study could provide a better understanding of choosable rice lines for human consumption and also as germplasm resources for future rice improvement programs.

## 1 Introduction

Rice (*Oryza sativa* L.) is one of the most widely cultivated crops including those with a pigment called coloured/pigmented rice. Rice, as a staple food crop, plays a very important role not only in supplying the nutrients and calories but also provide antioxidants needed to the world's human population to keep them healthy and for neutralizing free radicals and other reactive oxygen species (ROS). Free radicals and non-radical ROS, such as, hydrogen peroxide, hypochlorite, singlet oxygen, hydroxyl and superoxide, and nitric oxide radicals are highly reactive species that can harm cells and body functions. Antioxidants are stable molecules that donate an electron to neutralize free radicals, thus reducing cellular damage caused by free radicals (Lobo et al., 2010). Various compounds possessing antioxidant activities have been identified in rice, such as phenolics (phenolic acids like p-coumaric, ferulic, caffeic and syringic acids, and flavonoids such as flavonols, flavones, catechins and anthocyanins), vitamin E (tocopherols, tocotrienols and γ-oryzanol) and carotenoids (β-carotene, β-cryptoxanthin, lutein and zeaxanthin) (Iqbal et al., 2005; Zhang et al., 2010). Interestingly, the black and red-coloured rice cultivars are reported to contain higher amounts of antioxidant compounds such as polyphenols, anthocyanin, etc. compared to white and brown rice. These antioxidant compounds also possess anti-oxidative, anti-inflammatory, anti-diabetic, anti-microbial, anti-obesity and anti-carcinogenic activities (Abdel-Aal et al., 2006; He et al., 2011; Chen et al., 2012).

Another important constituent of rice are micronutrients, although they are contained in low quantity compared to other cereal crops, still, rice serves as the main source of nutrients for the majority of the Asian population (Sebastian and Prasad, 2015). Malnutrition such as energy malnutrition and micronutrient deficiencies has been a serious problem in developing countries. About 53% of all deaths of children under 5 years of age are associated with malnutrition (Muller and Krawinkel 2005). Hence, increasing the productivity of nutrient-rich rice using high yield varieties (HYV) technologies to meet the energy needed is not only required but also essential to deliver all the essential nutrients (Bouis and Welch, 2010).

Characterization of some pigmented rice of NE India has been attempted before (Saikia et al., 2012; Samyor et al., 2016). However, the number of rice cultivars and geographical area represented were limited. Hence, to substantiate the knowledge base of rice cultivars of the region, the present study was undertaken on the estimation of antioxidant (enzymatic and non-enzymatic), mineral and protein contents, and identification of functional groups using FT-IR of eleven cultivars (pigmented and non-pigmented) to facilitate their utilization for better human health.

## 2 Materials and methods

### 2.1 Plant samples

Pigmented and non-pigmented rice cultivars (black, red and white) were collected from 5 States of NE India during November and December of 2019 and 2020 (Table 1).

**Table 1:** Details of rice cultivars used in the study.

Sl. No.	Cultivar name	Colour	Collection	Geographical Coordinates
1.	Chakhao akupi	Black	Manipur	24° 29′ 33.7″ N
				93° 57′ 32.4″ E
2.	Chakhao poireiton	Black	Manipur	24° 29′ 33.7″ N
				93° 57′ 32.4″ E
3.	Lumre	Red	Nagaland	25° 39′ 04.8″ N
				94^0^ 01′ 13.9″ E
4.	Tsulu tsuk	Red	Nagaland	25° 57′ 40.6″ N
				94^0^ 02′ 24.6″ E
5.	Aamda	Red	Arunachal Pradesh	27° 56′ 20″ N
				95° 16′ 40.7″ E
6.	Tasung	Red	Arunachal Pradesh	27° 56′ 20″ N
				95° 16′ 40.7″ E
7.	Kawnglawng	Red	Mizoram	23° 28′ 06.1″ N
				93° 20′ 17.2″ E
8.	Fazu	Red	Mizoram	27° 56′ 20″ N
				95° 16′ 40.7″ E
9.	Menil mibabaret	Red	Meghalaya	25° 41′ 04.3″ N
				91° 54′ 46.9″ E
10.	Idaw	White	Mizoram	24° 15′ 40.5″ N
				92° 38′ 55.4″ E
11.	Farel	White	Mizoram	23° 58′ 29.6″ N
				93° 12′ 54.6″ E

NE states where rice seeds were collected.

### 2.2 Methanol extract preparation

The samples were extracted by using the modified method described by Walter et al. (2013). One gram seed powder was mixed with 20 ml of 80% methanol in a 50 ml tube and agitated for 1 h at room temperature, followed by centrifugation at 3000 rpm for 10 min. The supernatant was then collected. To the remaining residue, 20 ml of 80% methanol (pH 2) was added; and agitated, centrifuged and separated as described above. Then, to the remaining residue, 20 ml of 70% acetone was added followed by agitation, centrifugation and supernatant collection. The three resulting supernatants were pooled and used for further analyses.

### 2.3 Total phenolic content (TPC)

TPC was quantified by using Folin-Ciocalteu reagent as described by Iqbal et al. (2005). Briefly, 80 μl of extract was mixed with 2 ml of distilled water and 200 μl of 0.25 N Folin-Ciocalteu reagents. After 3 min, 1 ml of 7.5 % Sodium Carbonate was added and incubated in dark for 2 h at room temperature. Absorbance was then measured at 765 nm. The blank was prepared simultaneously containing methanol instead of extract. Gallic acid was used as standard and the results were expressed as mg GAE/100g grain (dry weight basis).

### 2.4 Total flavonoid content (TFC)

TFC was measured following the method described by Lin and Tang (2006). To 0.5 ml of extract, 1.5 ml of 95% methanol, 0.1 ml of 10% Aluminium Chloride, 0.1 ml of 1M potassium acetate and 2.8 ml of distilled water were added. The solution mixture was incubated for 40 min at room temperature. Absorbance was then measured at 415 nm. A quercetin standard curve was used to express the results as mg QE/100g dry matter.

### 2.5 Total anthocyanin content (TAC)

TAC was measured by using the method described by Abdel-Aal and Hucl (1999). The crude extract (20 μl) was mixed with 2ml of acidified methanol (methanol and 1N HCl, 85:15 v/v, pH1). Then, the absorbance was read at 535 nm against reagent blank. Cyanidin 3-Glucoside was used as a standard. TAC was calculated as Eq. (1):

(1) TAC (μg/g) = (A/ε) × (vol/1,000) × MW × (1/sample wt) × 10^6^

where A is absorbance, ε is molar absorptivity of Cyanidin 3-Glucoside, vol is the total volume of anthocyanin extract, and MW is the molecular weight of Cyanidin 3-Glucoside.

### 2.6 Total antioxidant activity (TAA)

TAA was determined by using the method described by Jan et al. (2013). To 300 μl extract, 3 ml Phosphomolybdate reagent (0.6 M Sulfuric acid, 4 mM Ammonium Molybdate and 28 mM Sodium Phosphate) was added. The tube was covered and incubated at 95 °C for 90 min and then cooled to room temperature. Then the absorbance was measured at 765 nm. The Ascorbic acid standard curve was used and the results were expressed as mg AAE/100g.

### 2.7 DPPH radical scavenging assay

DPPH radical scavenging was measured using the modified method described by Umnajkitikorn et al. (2013). Briefly, 100 μl of the extract was taken and mixed with 400 μl of 0.3 M Acetate buffer (pH 5.5) and 2.5 ml of 0.12 mM DPPH in 95% methanol. The mixture was incubated for 10 min in dark. Absorbance was measured at 517 nm. The radical scavenging activity was calculated using Eq. (2):

(2) DPPH radical scavenging activity (%) = A_b_ – A_s_/A_b_ x 100

where A_b_ is the absorbance of the control (using 80% methanol instead of the sample) and A_s_ is the absorbance of the sample.

### 2.8 Enzyme extraction

Enzyme extraction was performed according to Sunohara and Matsumoto (2004) with slight modification. To 0.5g of rice powder, 5 ml of extraction buffer (25 mM Potassium Phosphate buffer (pH 7.8), 0.4 mM EDTA, 1 mM Ascorbic acid and 2% PVPP) was added and the mixture was then centrifuged at 15000g for 20 min at 4 °C. The supernatant was filtered using Whatman No. 1 filter paper and the filtrate was collected for further analyses for enzyme activity.

### 2.9 CAT activity

Catalase (CAT) activity was determined by using the method described by Sunohara and Matsumoto (2004). A 1.9 ml of 25 mM H_2_O_2_ in 50 mM Potassium Phosphate buffer (pH 7) was added to 0.1 ml of enzyme extract. Then, the absorbance was measured at 240 nm in a UV-VIS spectrometer (Biospectrometer, Eppendorf, Germany). H_2_O_2_ was used as standard and the enzyme activity was defined as the amount of H_2_O_2_ (mM) decomposed per minute.

### 2.10 APX activity

Ascorbate Peroxidase (APX) activity was determined based on the method described by Sunohara and Matsumoto (2004). A 0.1 ml of enzyme extract was mixed with 0.5 ml of 100 mM Potassium Phosphate buffer (pH 7), 0.5 ml of 1 mM Ascorbic acid, 0.5 ml of 0.4 mM EDTA, and 0.02 ml of 10 mM H_2_O_2_. The absorbance was recorded at 290 nm. The H_2_O_2_ was used as standard and the enzyme activity was defined as the amount of H_2_O_2_ (mM) decomposed per minute.

### 2.11 SOD activity

Superoxide dismutase (SOD) activity was assayed based on the method described by Dhindsa et al. (1981). The assay mixture (3 ml) contained 0.1 ml of 1.5 M Sodium Carbonate, 0.1 ml of 3 mM EDTA, 0.2 ml of 200 mM methionine, 0.1 ml of 2.25 mM NBT, 1.5 ml of 100 mM Potassium Phosphate buffer, 0.95 ml distilled water and 0.5 ml of enzyme. The tube without enzyme extract was used as a control. The reaction was started by adding 0.1 ml riboflavin (60 uM) then the tubes were placed below a light source for 15 min. The reaction was stopped by switching off the light and the tubes were covered with aluminum foil. Finally, absorbance was measured at 560 nm and one unit of enzyme activity was defined as the quantity of enzyme which reduced the absorbance reading of the samples by 50% in comparison with control.

### 2.12 Total protein content

Total protein content was determined using Lowry's method (Lowry et al., 1951) and the results were expressed on a percentage basis.

### 2.13 Elemental content

Essential elements- Zinc (Zn), Iron (Fe), Calcium (Ca), Nickel (Ni) and Manganese (Mn) contents were estimated using Atomic Absorption Spectrophotometer (AAS, Shimadzu AA-7000) following standard protocols. Briefly, dehusked rice seeds were made to a fine powder using a pestle and mortar. A 0.1 g of sample was placed in a 100 ml conical flask then 20 ml of Nitric acid (HNO_3_) was added. The mixture was kept in a hot plate until the fuming of nitrogen dioxide ceased. Another 20 ml of HNO_3_ was added to the reaction and kept heating at a higher temperature. Then, hydrogen peroxide (H_2_0_2_) was added to the reaction to make the solution colourless. The mixture was heated until the solution was reduced to 3–5 ml. Then, the extract was diluted with 20 ml distilled water and filtered using Whatman filter paper. Finally, the sample was measured in an AAS. The results were expressed in μg/g.

### 2.14 Aroma test

The aromatic character was tested following the method described by Sood and Siddiq (1978). Briefly, 1 g of seed powder was placed on a Petri dish and 5 ml of 1.7% KOH solution was added. After 30 min, the dish was opened and smelling scored the presence or absence of aroma. The Institutional Human Ethics Committee, Mizoram University approved the experiment and informed consent was obtained from all participants for the sensory experiment.

### 2.15 FT-IR analysis

Functional groups present in studied rice grains were detected using a Fourier transformed infrared (FT-IR) spectroscopy. Rice flour mixed with Potassium Bromide (FT-IR grade) was pressed to form pellets and then fed into the FT-IR system (IRAffinity-1S, Shimadzu, Japan) in the frequency ranging from 400- 4000 cm^−1^.

### 2.16 Statistical analysis

The data was represented as mean ± standard deviation. The Pearson's correlation coefficient (r) for Antioxidant properties and Mineral content was performed using Graph Pad Prism Version 5 and Principal Component Analysis (PCA) of the FT-IR peaks was performed using OriginPro 2018.

## 3 Results and discussion

### 3.1 Antioxidant properties

The antioxidant activities of pigmented and non pigmented rice of NE India are presented in Table 2. Phenolic compounds are bioactive compounds that possess high anti-cancer, anti-viral, antioxidant and anti-bacterial potentials. In our analysis, phenolic content ranged from 94.8 to 900.90 mg GAE/100 g of dry weight. Among the studied cultivars, Lumre and Idaw possessed the highest and the lowest TPC respectively. These values were higher than the previous reports of 39–579, 142–262 and 53.47–80.16 mg GAE/100 g TPC of pigmented rice of NE India (Pathak et al., 2016; Saikia et al., 2012; Samyor et al., 2016). But the TPC was found to be much higher than another report on black rice of Brazil (Pedro et al., 2016). Total flavonoid content ranged from 3.46 to 286.76 mg QE/100 g of dry weight. Menil mibabaret possessed the highest total flavonoid content while Idaw had the lowest flavonoid content. Our study also showed that TFC was higher than that of a study on pigmented glutinous rice of NE India (45.57–60.76 mg QE/100 g TFC) by Pathak et al. (2016). Similarly, it was also higher than those reported (26.5–220.5 mg QE/100 g TFC) by Saikia et al. (2012). Flavanoids have high antimicrobial, anticancer, anti-inflammatory and anti-allergic potentials due to their ability to scavenge reactive oxygen species (ROS) consisting of free radicals (Montoro et al., 2005). Anthocyanin also possesses high antioxidant potential, antibacterial properties and is also regularly used as natural food colourants. Total anthocyanin content varied from 0.23 to 93.52 mg/100 g with Chakhao Poireiton showing the highest total anthocyanin content. The TAC was higher than a previous study on Canadian rice cultivars and black rice of Brazil (Abdel-Aal et al., 2006; Pedro et al., 2016). High values of TAC in Chakhao Poireiton and Chakhao Akupi were also comparable with a previous study by Asem et al. (2015). Interestingly, TAC values were much higher in the present study compared to the pigmented rice cultivars of Arunachal Pradesh in India (11.47–12.79 mg/100 g) (Samyor et al., 2016). Antioxidants from food have been gaining favourable attention owing to their significant roles in maintaining human health such as diseases prevention through inhibiting free radical formation. In our study, total antioxidant activity ranged from 2.92 to 86.74 mg AAE/100 g of dry weight. Lumre showed the highest antioxidant activity while Farel had the lowest antioxidant potentials. TAA was also slightly higher in the current study compared to a previous study (37.19–68.83 mmol TE/g TAA) on rice of Rio Grande do Sul state of Brazil (Walter et al., 2013). The high antioxidant potentials of the studied rice cultivars might be attributed to the high total phenolic and flavonoid presence.The DPPH radical scavenging activity ranged form 30.0 to 97.69%. Kawnglawng possessed the highest DPPH radical scavenging activity while Idaw had the lowest DPPH radical scavenging activity. DPPH radical scavenging activity also showed that pigmented rice under current investigation possessed higher scavenging activities compared to another study by Pathak et al. (2016) on pigmented glutinous rice of NE India (14.54%–21.73% DPPH respectively). Similarly, higher scavenging activity was also observed in our study compared to Asem et al. (2015) and Pedro et al. (2016). Then, similar radical scavenging activity was also observed in a study by Saikia et al. (2012) on some pigmented rice of NE India. Hence, the pigmented rice of NE India possessed high TPC, TFC, TAC, TAA and DPPH radical scavenging activity and could significantly improve human health. Plants possess anti-oxidative enzymes and non-enzymatic compounds that can protect themselves against oxidative damage caused by reactive oxygen species (ROS) (Mittler 2002). CAT, APX and SOD are enzymes that help in detoxifying ROS. For CAT activity, H_2_O_2_ decomposed per minute ranged from 0.11 mM to 5.25 mM. Tasung decomposed 5.25 mM H_2_O_2_ per minute. Aamda and Fazu decomposed the least amount of H_2_O_2_ per minute among the studied cultivars, followed by Chakhao poireiton and Tsulu tsuk (both decomposed 0.22 mM H_2_O_2_ respectively). For APX activity, H_2_O_2_ decomposed per minute ranged from 0.55 mM to 5.58 mM. Chakhao akupi decomposed 5.58 mM H_2_O_2_ per minute showing the highest activity of APX. Tsulu tsuk showed the lowest APX activity compared to other cultivars. And SOD activity ranged from 0.046 U to 0.39 U. Menil mibabaret possessed the highest SOD enzyme activity while Farel possessed the lowest SOD enzyme activity. We now can summarize that pigmented rice of NE India are good sources of CAT, APX and SOD and can significantly reduce stress oxidative reactions.

**Table 2:** Antioxidant activities of pigmented rice of NE India.

Cultivar name	Total Phenolic Content (mg GAE/100 g)	Total Flavanoid Content (mg QE/100 g)	Total Anthocyanin Content (mg/100 g)	Total Antioxidant Activity (mg AAE/100 g)	DPPH Radical Scavenging (%)	Catalase (mM H_2_O_2_ decomposed per minute)	APX (mM H_2_O_2_ decomposed per minute)	SOD (Unit)
Chakhao akupi	462.75 ± 9.87	133.47 ± 0.59	47.38 ± 1.59	49.27 ± 0.49	90.37 ± 3.76	1.09	5.58	0.36
Chakhao poireiton	662.86 ± 5.99	211.68 ± 0.14	93.52 ± 1.59	63.02 ± 0.34	93.45 ± 1.5	0.22	5.14	0.06
Lumre	900.90 ± 2.26	214.18 ± 13.15	13.25 ± 3.93	86.74 ± 0.35	60.1 ± 0.76	0.98	0.66	0.11
Tsulu tsuk	572.61 ± 5.99	196.97 ± 1.75	8.58 ± 0.60	70.29 ± 0.29	55.18 ± 0.9	0.22	0.55	0.17
Aamda	487.60 ± 3.92	109.91 ± 0.88	7.99 ± 1.04	52.89 ± 0.29	52.23 ± 0.54	0.11	1.75	0.11
Tasung	534.68 ± 6.80	33.47 ± 7.89	4.08 ± 1.59	47.98 ± 0.51	86.41 ± 0.26	5.25	5.47	0.16
Kawnglawng	784.49 ± 4.53	278.25 ± 1.02	32.75 ± 1.59	60.88 ± 1.85	97.69 ± 0.49	0.77	1.09	0.29
Fazu	646.32 ± 4.99	241.29 ± 4.24	14.8 ± 1.59	58.29 ± 0.89	95.73 ± 1.25	0.11	2.19	0.2
Menil mibabaret	817.19 ± 3.92	286.76 ± 0.44	19.3 ± 3.74	63.35 ± 1.01	87.6 ± 1.17	0.33	3.72	0.39
Idaw	94.8 ± 15.46	3.46 ± 0.46	0.27 ± 0.016	5.07 ± 1.32	30.0 ± 5.5	0.65	0.62	0.06
Farel	287.66 ± 16.33	4.48 ± 0.46	0.23 ± 0.023	2.92 ± 0.67	34.29 ± 0.49	0.58	0.84	0.046
Mean	568.35 ± 70.1	155.81 ± 31.81	22.08 ± 8.34	50.97 ± 7.7	71.86 ± 7.66	1.01	2.91	0.2

Our study also supports the previous findings of high antioxidant contents in pigmented rice than the non-pigmented ones. Hence, the presence of natural antioxidants in a higher proportion among the pigmented rice of NE India can be suggested for low cost, highly compatible dietary intake with no harmful effects for human consumption. Likewise, an inverse relationship was also reported between the dietary intake of antioxidant-rich food and the incidence of human diseases (Lobo et al., 2010). Then, a correlation analysis was performed among the antioxidant activities (TPC, TFC, TAC, TAA and DPPH) where a significant correlation between TPC and TAA (r = 0.900) was observed at a 95% confidence level followed by TPC and TFC (r = 0.866) (Table 4).

### 3.2 Total protein content

The total protein contents ranged from 3.62% to 11.06% with an average of 8.18% (Table 3). Chakhao poireiton possessed the highest total protein content followed by Tsulu tsuk, Aamda, Menil mibabaret etc. and Farel possessed the lowest among the studied cultivars. The average protein content of the pigmented cultivars was higher than the previous reports on pigmented rice of NE India and a majority of the studied pigmented cultivars possessed higher protein content than the previous studies (Saikia et al., 2012; Samyor et al., 2016). The differences in protein content between rice accessions could be due to a variety of factors including water supply, handling, fertiliser application (soil nitrogen availability), environmental stress (such as salinity and alkalinity, temperatures, and diseases), growing area location, growing conditions, and time, all of which tend to increase grain protein content (Buresova et al., 2010). Hence, pigmented rice as a good source of proteins is well justified in the current investigation.

**Table 3:** Mineral, protein and aromatic characteristics of pigmented rice of NE India.

Cultivar name	Zn (μg/g)	Fe (μg/g)	Ca (μg/g)	Ni (μg/g)	Mn (μg/g)	Protein content (%)		Aroma
Chakhao akupi	33.46	131.08	544.04	108.92	43.2	9.4		Strong
Chakhao poireiton	65.38	134.56	790.68	177.24	85.2	11.06		Strong
Lumre	58.54	53.96	527.72	57.12	55.6	9.1		Mild
Tsulu tsuk	70.74	36.8	444.3	52.32	80.56	11.02		Mild
Aamda	46.04	61	424.8	42.44	73.84	10.34		Mild
Tasung	42.9	34.92	953.88	44.32	37.66	9.06		Mild
Kawnglawng	38.3	212.64	377.2	34.7	36.62	6.06		Strong
Fazu	41.32	252.62	714.5	150.28	50.82	7.24		Mild
Menil mibabaret	28.64	49.82	643.78	31.5	30.94	9.42		Strong
Idaw	0.63	1.45	1.12	0.14	0.35		3.72	Mild
Farel	0.49	1.24	1.47	0.1	0.28		3.62	Mild
Mean	38.76	88.19	493.04	63.55	45.00		8.18

**Table 4:** Correlation coefficient among the antioxidant activities.

Antioxidant activities	Total Flavanoid Content	Total Anthocyanin Content	Total Antioxidant Activity	DPPH Radical Scavenging
Total Phenolic Content (TPC)	0.865 (0.0006)	0.311 (0.35)	0.900 (0.0002)	0.649 (0.03)
Total Flavanoid Content (TFC)		0.416 (0.202)	0.800 (0.003)	0.665 (0.025)
Total Anthocyanin Content (TAC)			0.330 (0.32)	0.589 (0.056)
Total Antioxidant Activity (TAA)				0.578 (0.06)

(Values in parentheses indicate the p-values).

### 3.3 Mineral content

The mineral contents of black, red and white rice of NE India are presented in Table 3. Ca, Zn, Fe, Ni and Mn contents of rice cultivars were estimated. Zn content ranged from 0.49 to 70.74 μg/g. Tsulu tsuk showed the highest Zn content while Farel had the lowest Zn content among the studied cultivars. Fe content ranged from 1.24 to 252.62 μg/g. Fazu possessed the highest Fe content and Farel possessed the lowest Fe content. Ca content ranged from 1.12 to 953.88 μg/g. Tasung had the highest Ca content and Idaw had the lowest Ca content. Ni content ranged from 0.1 to 177.24 ug/g. Chakhao poireiton possessed the highest Ni content. Farel had the lowest Ni content. Mn content ranged from 0.28 to 85.2 μg/g. Chakhao poireiton possessed the highest Mn content while Farel possessed the lowest Mn content. Fe content in the present study was higher and Zn content was comparable (6–16 μg/g Fe and 17–59 μg/g Zn) with Thai brown rice varieties (Saenchai et al., 2012). The averages of Zn, Mn, and Fe contents were higher (34.8 μg/g Zn, 29.9 μg/g Mn, 92.7 μg/g Fe) than the previous study of local rice germplasm of Tripura, India (Dikshit et al., 2016). Overall, Fe and Zn contents were also higher (0.25–34.8 μg/g Fe and 0.85–195.3 μg/g Zn) than rice varieties of West Bengal and adjoining areas of India (Roy and Sharma 2014). Then, a correlation analysis was performed among the mineral elements (Zn, Fe, Ca, Ni and Mn). Again, a significant correlation between Zn and Mn (r = 0.944) was observed at a 95% confidence level (Table 5).

**Table 5:** Correlation coefficient among the mineral elements.

Elements	Fe	Ca	Ni	Mn
Zn	0.294 (0.38)	0.659 (0.027)	0.564 (0.07)	0.943 (0.0001)
Fe		0.386 (0.24)	0.670 (0.023)	0.309 (0.38)
Ca			0.636 (0.035)	0.580 (0.027)
Ni				0.630 (0.037)

(Values in parentheses indicate the p-values).

Mineral content in rice is affected by genetic traits of accessions and environmental conditions (Zimmermann and Hurrell, 2002). Jiang et al. (2007) have stated that genotypic differences may provide possibilities to select for rice germplasm with greater mineral elements. According to Heinemann et al. (2005), identifying the natural genetic variability of the elements is critical because an increase in mineral content would contribute significantly to the Recommended Dietary Allowances (RDA). Our analysis showed the presence of essential nutrients that are required for metabolic processes like respiration and DNA synthesis. So, the findings suggest the effective utilization of these cultivars as a source of minerals or nutrient supplement. Further, our investigation supports a previous report of high mineral content among the rice cultivars of NE India that can be used for designing crop improvement programs for better human health (Vanlalsanga et al., 2019).

### 3.4 Aromatic characteristics

The aromatic characteristics of the rice cultivars are presented in Table 3. All the studied rice cultivars possessed aromatic characteristics. Chakhao akupi, Chakhao poireiton, Kawnglawng and Menil mibabaret possessed a strong aroma while Lumre, Tsulu tsuk, Aamda, Tasung, Fazu, Idaw, Farel possessed mild aromatic characteristic.

### 3.5 FT-IR analysis

**Figure 1:**
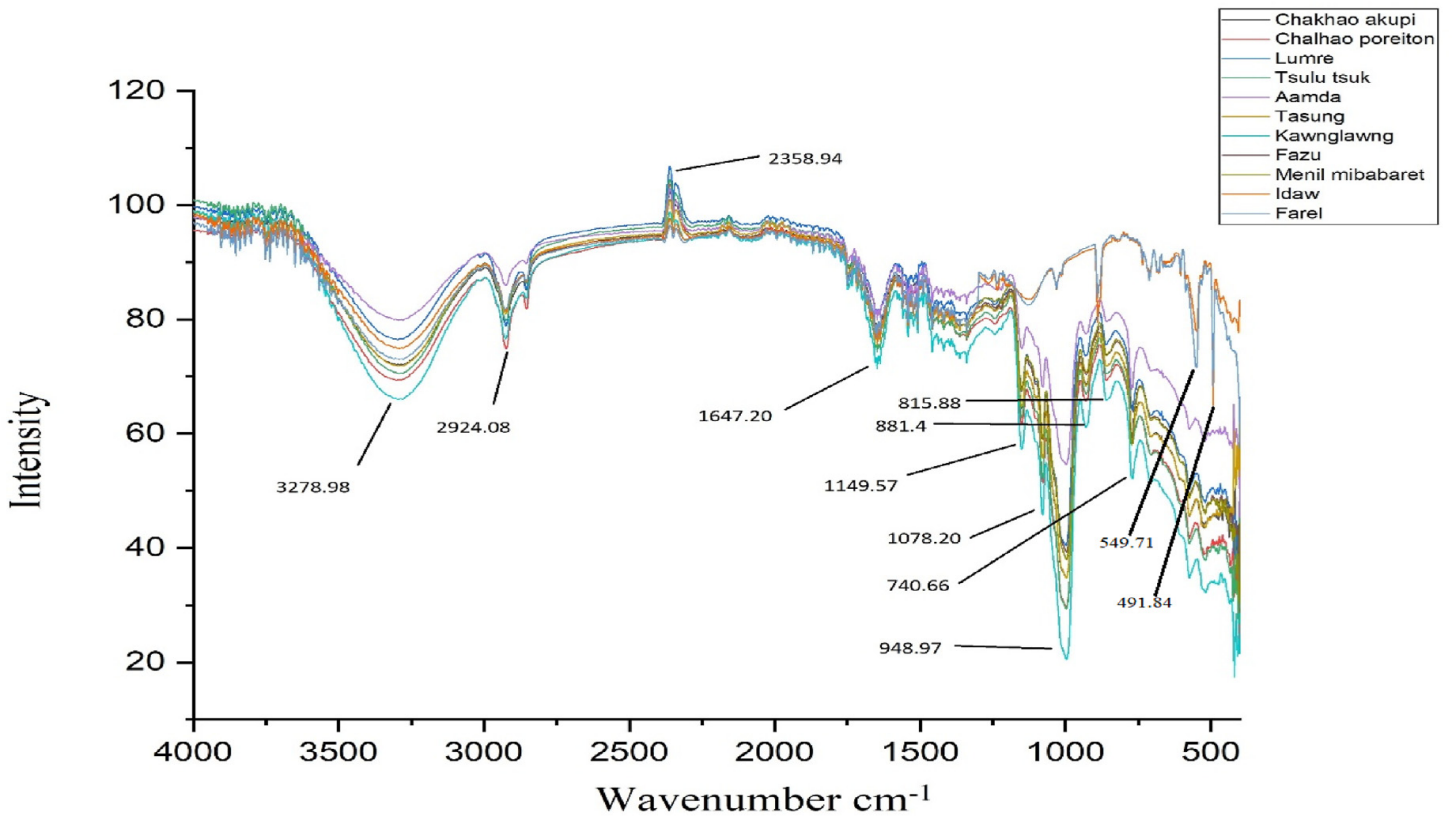
Representative FT-IR average spectra of rice used in the study.

Using FTIR, we were able to determine the functional groups found in rice grain, which will aid in the identification of chemical composition, elucidation of chemical structure, and comprehension of the role of functional groups as bioactive molecules in phytopharmaceutical formulations and nutritional composition. FTIR spectra of different rice varieties are presented in Figure 1. The spectra pattern between the wavenumbers 1500-4000 cm^−1^ were found to be analogous among the studied rice varieties (Table 6). A strong and broad peak in the region 3800-3000 cm^−1^ represents O–H stretching vibration absorption overlapping with N–H stretching vibration (Wei et al., 2021). An earlier study also reported the presence of peaks at the region between 3520-3320 nm, which correspond to the aromatic amines (Samyor et al., 2016). The peak at 2924 is associated with the asymmetric stretching vibration of methylene (Wei et al., 2021). The absorption peaks between the wavenumbers of 1800–750 cm^−1^ represent the fingerprint region, which has a unique characteristic pattern for a particular sample. The bands in this region correspond to the presence of carbohydrates, lipid, protein secondary structures, and polyphenols. An earlier study also reported the presence of bands in the region of 1700 to 1200 cm^−1^ that were associated with the minor functional components (protein, lipid) in rice flour (Warren et al., 2016). Correspondingly, two peaks (1647 and 1257 nm) were observed in this region in our study. The FTIR bands at the region of wavenumbers 1300-900 cm^−1^ correspond to the C–O stretching/C–C stretching/C–O–C stretching in starch and lipid (Wei et al., 2021). In the present study, three prominent peaks at 1149, 1078, and 948nm were found in this region. Peaks at 881 and 815 nm correspond to the C–C stretching (Ma et al., 2018). And peaks also appeared at 740 and 680 corresponds to the = CH bending similar to Trivedi et al. (2015). Furthermore, functional groups identification through FTIR analysis can be correlated with its importance in a variety of pharmaceutical goods, including anti-cancer, anti-ulcer, jaundice, headache, stomach ache, and anti-inflammatory medications, as well as sources of antibacterial and antioxidant substances (Baker 1982; Skoog et al., 2007; Maobe and Nyarango 2013). The present study also revealed that the intensity of peaks is different among the studied rice varieties. It was observed that the coloured rice had higher peak intensity compared to that of the white rice. And FT-IR spectroscopy was found to be a reliable method to characterize and evaluate the functional groups of antioxidant compounds present in rice samples.

**Table 6:** Assignment of main bands of the FT-IR spectra of rice samples.

Frequency (nm)	Functional group	References
3278	-O-H/-N-H stretching	Wei et al., (2021)
2924	-C-H stretching	Wei et al., (2021)
2358	O=C=O stretching	Ferreira et al., 2020
1647	C=O asymmetric stretching	Trivedi et al., (2015)
1149, 1078, 948	C–O stretching/C–C stretching/C–O–C stretching	Samyor et al., (2016); Qiu et al., (2021)
881, 815	C–C stretching	Ma et al., (2018)
740, 680	= CH bending	Trivedi et al., (2015)
549, 491	C–I (Halogen compound)	Lingegowda et al., (2012)

### 3.6 Principal Component Analysis (PCA)

**Figure 2:**
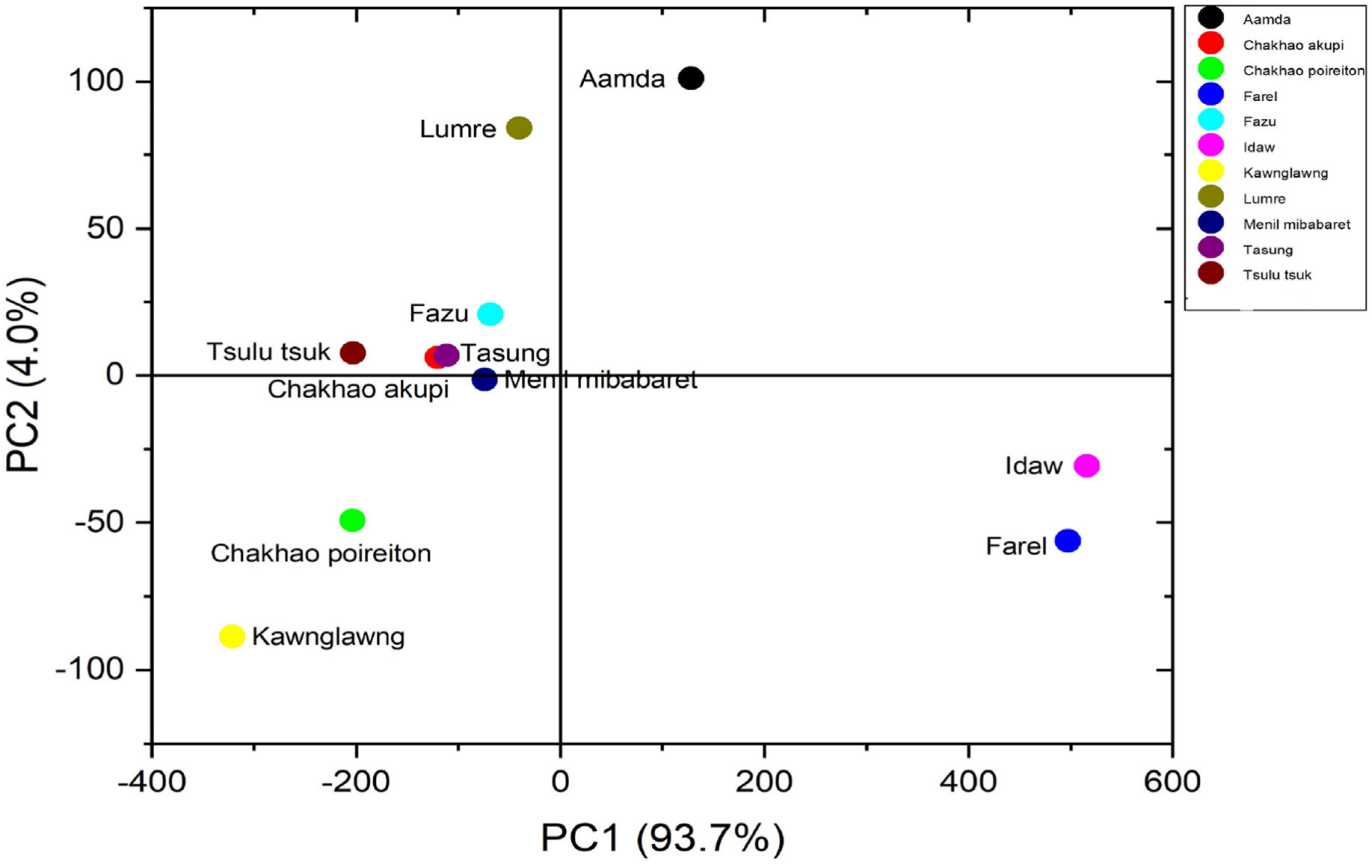
Principal Component Analysis (PCA) based on FT-IR spectra.

Principal Component Analysis (PCA) is a powerful tool for the analysis of data and simplifies the complexity of large data that can be represented as summaries. A PCA plot was developed using the FTIR peaks from wavenumber 400–4000 nm. Figure 2 shows the scattered plot associated with PC1 and PC2. PCA and percentage contribution of each component to the total variation showed that the first principal component contributed 93.7% and the second principal component contributed 4.0%. Kawnglawng and Chakhao poreiton were found in the region of negative PC1 and negative PC2, Fazu, Tsulu tsuk, Tasung, Chakhao akupi, Menil mibabaret and Lumre in the region of negative PC1 and positive PC2, and Aamda in the region of positive PC1 and positive PC2. Idaw and Farel were found in the region of positive PC1 and positive PC2. The PCA scattered plot revealed that the separation occurred due to the similarity of the functional groups and dispersion of the same samples are due to the dissimilarities in absorption intensity. The main differences were seen in the fingerprint region and the most important loadings (peaks) in the separation were 948, 549 and 491 cm^−1^ (da Silva et al., 2017; Qiu et al., 2021). Moreover, the non pigmented cultivars were found separated from the pigmented cultivars. Hence, rice cultivars under current investigation possessed diverged biologically active functional groups. These functional groups can be effectively used in different pharmaceutical formulations viz. anti-cancers, anti-ulcers, jaundice, headache, stomach ache and anti-inflammatory drugs; or as sources of antioxidant, antimicrobial compounds etc (Maobe and Nyarango 2013). Pigmented rice provides more health benefit than white rice and it contains higher amounts of phenolic, flavonoid and anthocyanin contents compared to white rice. Chanu et al. (2016) also opined that black rice cultivars are good sources of protein, Zn, Fe, Mn and antioxidant compounds. Thus, our finding on the pigmented rice of NE India also satisfies the previous findings.

It is also interesting to note that farmers in some areas of NE India practice shifting cultivation sowing indigenous varieties, not only rice but also other crops, so these hill areas serve as conservation field for landraces (Vanlalsanga and Singh 2019). The introduction of high yielding varieties results in the loss of diversity of indigenous cultivars due to differential cultivation practices.

## 4 Conclusions

Nutrients and antioxidants are vital for human metabolic processes. In order to receive enough nutrients and antioxidant compounds, it is very important to characterize rice varieties based on minerals and antioxidant compositions, especially in Asia where rice is the main staple food. So, the present investigation can provide a better understanding of pigmented rice cultivars of NE India regarding their antioxidant and mineral contents. Furthermore, it can be concluded that northeast India possess potential rice cultivars that can be used as health and nutritional supplement and also as important ingredient of food products. This study will also provide choosable rice for daily consumption for the betterment of human health and also germplasm resources for future rice improvement. Management, utilization and conservation of indigenous rice need to be considered seriously because these landraces possessed high genetic diversity and genetically considerable traits, which are good sources for future rice management and improvement.

## Declarations

### Author contribution statement

Sagolshem Priyokumar Singh: Performed the experiments; Analyzed and interpreted the data; Wrote the paper.

Vanlalsanga: Performed the experiments; Analyzed and interpreted the data; Wrote the paper.

S.K. Mehta: Analyzed and interpreted the data; Wrote the paper.

Y. Tunginba Singh: Conceived and designed the experiments; Analyzed and interpreted the data; Contributed reagents, materials, analysis tools or data; Wrote the paper.

### Funding statement

This research did not receive any specific grant from funding agencies in the public, commercial, or not-for-profit sectors.

### Data availability statement

Data included in article/supp. material/referenced in article.

### Competing interest statement

The authors declare no conflict of interest.

### Additional information

No additional information is available for this paper.
